# Aging and the optimal viewing position effect in Chinese

**DOI:** 10.3389/fpsyg.2015.01656

**Published:** 2015-10-29

**Authors:** Pingping Liu, Danlu Liu, Buxin Han, Kevin B. Paterson

**Affiliations:** ^1^Key Laboratory of Mental Health, Centre on Aging Psychology, Institute of Psychology, Chinese Academy of SciencesBeijing, China; ^2^Department of Neuroscience, Psychology and Behaviour, College of Medicine, Biological Sciences, and Psychology, University of LeicesterLeicester, UK

**Keywords:** viewing position, eye movements, Chinese words, aging, word recognition

## Abstract

Substantial evidence indicates that where readers fixate within a word affects the efficiency with which that word is recognized. Indeed, words in alphabetic languages (e.g., English, French) are recognized most efficiently when fixated at their *optimal viewing position* (OVP), which is near the word center. However, little is known about the effects of fixation location on word recognition in non-alphabetic languages, such as Chinese. Moreover, studies to date have not investigated if effects of fixation location vary across adult age-groups, although it is well-established that older readers experience greater difficulty recognizing words due to visual and cognitive declines. Accordingly, the present research examined OVP effects by young and older adult readers when recognizing Chinese words presented in isolation. Most words in Chinese are formed from two or more logograms called characters and so the present experiment investigated the influence of fixation location on the recognition of 2-, 3-, and 4-character words (and nonwords). The older adults experienced generally greater word recognition difficulty. But whereas the young adults recognized words most efficiently when initially fixating the first character of 2-character words and second character of 3- and 4-character words, the older adults recognized words most efficiently when initially fixating the first character for words of each length. The findings therefore reveal subtle but potentially important adult age differences in the effects of fixation location on Chinese word recognition. Moreover, the similarity in effects for words and nonwords implies a more general age-related change in oculomotor strategy when processing Chinese character-strings.

## Introduction

The optimal viewing position (OVP) effect refers to the longstanding finding that where the eyes initially fixate within an isolated word influences how easily that word can be recognized. For words in alphabetic languages (e.g., English, French), the OVP is a little to the left of the center of words, and numerous studies show words in these languages are processed most efficiently when fixated at this location (O'Regan et al., 1984; Vitu et al., 1990; Brysbaert and Meyers, 1993; Farid and Grainger, 1996; Ducrot et al., 2013; Yao-N'Dre et al., 2013; Ferrand and Augustinova, 2014). This influence of fixation location on word recognition is observed in lexical decision latencies and naming times, which are typically shorter when words are initially fixated at the OVP than other locations. Moreover, studies including measures of eye movements show that participants make fewer refixations when words are initially fixated at the OVP (e.g., Vitu et al., 1990; Hyönä and Bertram, 2011; Ducrot et al., 2013; Liu and Li, 2013; Velan et al., 2013).

However, the underlying cause of the OVP effect is elusive. The rapid drop-off in retinal acuity that occurs with increasing eccentricity from the point of gaze even within central vision seems an especially important component of the effect (McConkie et al., 1989; Nazir, 1991; Nazir et al., 1992; Clark and O'Regan, 1999; Brysbaert and Nazir, 2005; Jordan et al., 2010; Hyönä and Bertram, 2011). But this constraint predicts that words are recognized most efficiently when fixated at their very center, as fixations at this location maximize the number of letters that project to high-acuity retinal regions. Consequently, acuity alone cannot explain why the OVP is to the left of center of words in languages read from left-to-right. This has led various researchers to propose additional constraints. These include perceptual learning, which proposes that the OVP is a consequence of words being recognized most efficiently when viewed from the same location that they are habitually fixated during natural reading (Farid and Grainger, 1996; Nazir et al., 1998, 2004; Brysbaert and Nazir, 2005; Wong and Hsiao, 2012). Fixations tend to be made at locations a little to the left of a word's center during the natural reading of languages like English and French, which Rayner (1979) referred to as the *preferred viewing location* (or PVL, see also McConkie et al., 1989; Paterson et al., 2012). Perceptual learning therefore correctly predicts that the OVP and PVL will be broadly similar for words in such languages. However, the PVL tends to be to the *right* of the center of words for languages read from right-to-left, such as Arabic and Hebrew (Pollatsek et al., 1981; Deutsch and Rayner, 1999; Paterson et al., 2015). Perceptual learning predicts that words in these languages will be recognized most efficiently when viewed from this location. However, studies show that the OVP is at the center of words in these languages (Farid and Grainger, 1996; Deutsch and Rayner, 1999; Jordan et al., 2011). Consequently, the contribution of perceptual learning to the OVP effect remains unclear. Accordingly, while the OVP may indicate the optimal position for word identification, the PVL represents the locations that are actually fixated in words during natural reading, and these need not be the same.

An alternative possibility is that words are recognized most efficiently when fixated at the region that is most informative about the meaning of the word (O'Regan, 1981; Farid and Grainger, 1996; Stevens and Grainger, 2003). According to this view, the OVP is to the left of word center for languages like English and French because the beginning letters are often highly informative about the meaning of words in these languages. By comparison, because Arabic and Hebrew have a non-concatenative morphology in which the morphological root that conveys the core meaning is usually distributed throughout a word, this may explain why the OVP is at the word center in these languages (Farid and Grainger, 1996; Deutsch and Rayner, 1999; Jordan et al., 2011). Studies have investigated the role of the informativeness of different parts of words by examining OVP effects for nonwords (i.e., orthographically legal pseudowords) and meaningless strings of letters or symbols, such as z-strings. However, while some studies show an OVP effect for nonwords (Hutzler et al., 2008; Liu and Li, 2013) and even letter-strings during mindless-reading (Nazir, 1991; Vitu et al., 1995; Nuthmann et al., 2007), others show no such effect (Auclair and Chokron, 2001; Nazir et al., 2004; Grainger et al., 2010; Ducrot et al., 2013). Consequently, the extent to which the OVP is influenced by the linguistic characteristics of the stimuli also remains to be more fully determined.

Finally, it is argued that asymmetries in cerebral hemispheric processing contribute to the OVP effect (Brysbaert, 1994; Brysbaert and Nazir, 2005; Jordan et al., 2011). The left hemisphere is specialized for word recognition for the vast majority of individuals (Cabeza, 2002; Daselaar and Cabeza, 2005). Moreover, because the right and left visual fields project contra-laterally to the two cerebral hemispheres, words presented to the right visual field benefit from direct access to the left hemisphere and so are recognized most efficiently. By comparison, words presented to the left visual field first project to the right hemisphere, which is inferior for word recognition, before being passed to the left hemisphere via inter-hemispheric transfer. As a consequence, linguistic stimuli presented to the left visual field are processed less efficiently (e.g., Iacoboni and Zaidel, 1996; Simola et al., 2009; Ducrot et al., 2013). Based on these hemispheric processing asymmetries, it has been proposed that the OVP is to the left of the word center in English and French because this facilitates word recognition by ensuring more letters in words fall in the right visual field and so project directly to the left hemisphere (Brysbaert and Meyers, 1993; Brysbaert, 1994; Brysbaert and Nazir, 2005). However, while it is argued that this division in hemispheric processing extends up to the point of fixation (e.g., Ellis and Brysbaert, 2010), this is not supported by experimental evidence (for a review, Jordan and Paterson, 2009) and processing appears functionally bilateral within a region at least 1° wide around the point of fixation. Consequently, hemispheric influences may only be observed for longer words, or particularly large stimuli, that project beyond this region into regions of unilateral hemispheric processing.

Until recently, OVP studies have focused on word recognition in alphabetic languages and by skilled young adult readers. But investigations of OVP effects in non-alphabetic languages and by different age-groups of readers will shed fresh light on the role of fixation location in word recognition. Chinese is particularly well-suited to assessing the universality of the OVP effect as it differs fundamentally from the alphabetic languages that have been the focus of research to date. Unlike these alphabetic languages, Chinese uses a logographic writing system in which words are formed from box-like pictorial symbols (i.e., logograms) called characters. According to the Chinese lexicon (2003), relatively few words correspond to a single character (3%) and most are formed by two (64%), three (18%), or four (14%) characters. However, the influence of fixation location on the recognition of Chinese words presented in isolation has received relatively little attention. Indeed, only one study to date, by Liu and Li (2013), has examined the OVP effect in Chinese. This study showed that readers recognized words most efficiently when they initially fixated the first character of 2-character words and the second character of 3- or 4-character words. The same pattern of effects was obtained for character strings that did not form words in Chinese, and so Liu and Li took this similarity in the OVP effect for words and nonwords to show that the advantage of fixating a particular character location reflects the normal oculomotor strategy of Chinese readers rather than a specific advantage for word recognition.

The aim of the present experiment was to extend this research by investigating if there are adult age differences in the OVP effect during Chinese word recognition. Older adults have particular difficulty recognizing words and tend to produce slower response than young adults in studies of isolated word recognition (Balota et al., 2004; Ratcliff et al., 2004; Stine-Morrow et al., 2006; Goral et al., 2008). Moreover, studies of natural reading show that older adults read more slowly than young adults and make more and longer fixations on words (Kemper et al., 2004; Rayner et al., 2006, 2014; Paterson et al., 2013; Payne et al., 2014). Moreover, older readers may also have a smaller and more symmetrical perceptual span (Rayner et al., 2009, 2010; but see Risse and Kliegl, 2011). These age-related changes in reading behavior are widely attributed to sensory and cognitive declines in older age and may lead older adults to adapt their oculomotor strategy to compensate for their generally poorer processing of text (Rayner et al., 2006, 2009; Paterson et al., 2013).

However, little is known about the effects of normal aging on the recognition of isolated Chinese words. Moreover, no studies to date (to our knowledge) have investigated age differences in the OVP effect even in alphabetic languages. However, visual and cognitive declines in older age may have an important influence on the recognition of multi-character words in Chinese. In particular, if older adults have greater difficulty identifying the component characters of a word in Chinese, due to reduced acuity or slower lexical processing, this may produce differences in the effects of fixation location on word recognition. One possibility is that older adults suffer more than young adults from fixating words at suboptimal character locations, in which case they may produce larger OVP effects. Alternatively, as a result of age-related difficulty recognizing words, older adults may produce qualitatively different effects of fixation location compared to young adults by, for example, employing a more cautious strategy in which they serially process characters that form words. However, the effects of healthy aging on word recognition in Chinese are entirely unknown, and so the present research undertook a novel assessment of adult age differences in the effects of fixation location on the recognition of Chinese words. As in the study by Liu and Li (2013), we examined effects for words and nonwords that varied in length (2-, 3-, or 4-characters). Some studies of natural reading have investigated initial landing positions on words in Chinese by dividing characters in two (i.e., the first and second half of a character; e.g., Yan et al., 2010). However, in order to ensure comparability with the findings from the word recognition study by Liu and Li, we examined the effects of fixating different possible character positions in our target words (rather than half-character positions). This enabled us to establish whether OVP effects differ for young and older adults and if the pattern of OVP effects produced by either age-group resembles that reported in this earlier study.

## Methods

### Participants

Twenty-four young adults (aged 18–33 years, *M* = 21.6 years) from universities near the Institute of Psychology in Beijing and twenty-four older adults (aged 60–83 years, *M* = 70.3 years) from the local Beijing community participated in the experiment. These two age groups were matched for years of formal education (young adults = 14.0 years, older adults = 13.5 years, *p* = 0.46). All participants were native Chinese speakers and had normal or corrected vision. All participants reported that they spent at least half an hour reading each day.

### Ethics statement

The study was approved by the Institutional Review Board of the Institute of Psychology, Chinese Academy of Sciences. All participants were provided informed consent before taking part in our experiments.

### Stimuli and design

Stimuli consisted of 360 words and 360 nonwords. Word stimuli were selected from the *Chinese Lexicon* (2003). Nonwords were constructed by combining randomly-selected Chinese characters; with the requirement that these did not form valid Chinese words and that no consecutive characters could form a word. This was confirmed by three young adults and three older adults (all native Chinese) who did not take part in the experiment. Stimuli were 2, 3, or 4 characters long. Each character within a stimulus served as an initial viewing position during the experiment, and there were 40 stimuli of each length for each viewing position. Thus, for 2-character stimuli there were 80 words and 80 nonwords, for 3-character stimuli there were 120 words and 120 nonwords, and for 4-character stimuli there were 160 words and 160 nonwords. Table 1 shows the frequency of words and the frequency and complexity of the characters at each location within the words and nonwords. For stimuli of each length, character frequency, and character complexity were matched (*ps* > 0.30).

**Table 1:** **Properties of the stimuli used in the study**.

	**Word frequency**	**Character frequency**					**Character complexity**				
		**First**	**Second**	**Third**	**Fourth**	***p***	**First**	**Second**	**Third**	**Fourth**	***p***
2-Character words	104	983	959			0.83	8.2	8.3			0.70
2-Character nonwords		956	980			0.90	8.7	8.8			0.95
3-Character words	3107	840	1025	818		0.36	7.9	8.0	8.2		0.56
3-Character nonwords		990	859	782		0.34	8.2	7.8	7.8		0.44
4-Character words	688	1029	1186	1126	1099	0.79	7.9	7.8	7.8	8.0	0.80
4-Character nonwords		967	1181	1128	1070	0.56	7.9	8.1	8.3	8.1	0.58

*Word and character frequency are in occurrences per million. The number of individual strokes in a character is treated as the index of character complexity*.

In total, 720 experimental trials were presented in random order to each participant following 36 practice trials. Although each participant viewed all the stimuli, the character that served as the initial viewing position for each stimulus was counterbalanced across participants, so that each participant viewed each stimulus only once but viewed an equal number of stimuli of each length at each viewing position.

### Apparatus and procedure

An EyeLink 1000 eye-tracker (SR Research, Osgoode, Canada) ensured accurate fixation at the designated fixation location on each trial and recorded eye movements. Although viewing was binocular, only right eye movements were recorded. Stimuli were presented on a 21-inch CRT monitor (1024 × 768 resolution, 150 Hz vertical refresh rate) as white text on a gray background. The contrast was intentionally low to prevent eye fatigue. Each stimulus was displayed in 26-point Song font, and the size of each character was 35 × 35 pixels. Participants were seated at a viewing distance of 58 cm from the computer monitor and at this viewing distance each character subtended approximately 1.3°.

Participants were tested individually. At the beginning of the experiment, each participant performed a 3-point calibration procedure, and we ensured that calibration was accurate to 0.5° of visual angle or lower. At the beginning of each trial, a white square (subtending approximately 0.9 × 0.9°) was displayed at the screen center. Once the participant fixated this square, it was replaced by two vertically-aligned line segments, separated by a gap equal in height to one character, which participants were instructed to fixate. After 500 ms, the vertical lines disappeared and a stimulus was presented with one of its characters replacing the gap. This stimulus remained visible until the participant made a lexical decision by pressing one of two keys on a response pad. Participants were instructed to respond as quickly and accurately as possible. The next trial began immediately after this response. For each participant, the experiment lasted approximately an hour.

## Results and discussion

Overall accuracy was high and did not differ between the young adults (96%) and the older adults [97%, *t*_(46)_ = 1.56, *p* = 0.13; see Table 2). Data from trials with incorrect responses (young adults, 4.7% of word trials, 4.2% of nonword trials; older adults, 4.0% of word trials, 2.7% of nonword trials) or trials with RTs three standard deviations above or below each participant's mean (young adults, 1.2% of word trials, 2.0% of nonword trials; older adults, 1.4% of word trials, 1.6% of nonword trials) were excluded from the analyses of RTs and eye movements. To reveal effects of viewing position on eye movements, we analyzed *refixation probability* (the probability that the stimulus was fixated more than once) and first *fixation duration* (the duration of the first fixation on the stimulus, regardless of the number of fixations). For each stimulus length, data were analyzed using a mixed-design analysis of variance (ANOVA) with age-group as a between-participants factor, and stimulus type and viewing position as within-participant factors. Furthermore, for each age group, we conducted individual ANOVAs and polynomial trends for each word and nonword length. In order to reduce false positives in reading experiments (von der Malsburg and Angele, 2015), Bonferroni correction was applied to these *post-hoc* pairwise comparisons between the different initial viewing positions if the main effects were significant.

**Table 2:** **Mean accuracy for the lexical decision task of all stimuli lengths on the imposed initial viewing positions for the older and young adults**.

**Stimuli length**		**Character 1**	**Character 2**	**Character 3**	**Character 4**
**WORD**					
2-Character	Older	0.98 (0.006)	0.97 (0.008)		
	Young	0.97 (0.006)	0.96 (0.008)		
3-Character	Older	0.97 (0.010)	0.96 (0.008)	0.95 (0.010)	
	Young	0.94 (0.010)	0.95 (0.008)	0.93 (0.010)	
4-Character	Older	0.96 (0.008)	0.95 (0.008)	0.96 (0.010)	0.96 (0.009)
	Young	0.96 (0.008)	0.98 (0.008)	0.96 (0.010)	0.94 (0.009)
**NONWORDS**					
2-Character	Older	0.92 (0.021)	0.95 (0.016)		
	Young	0.92 (0.021)	0.94 (0.016)		
3-Character	Older	0.99 (0.005)	0.99 (0.004)	0.99 (0.005)	
	Young	0.98 (0.005)	0.99 (0.004)	0.98 (0.005)	
4-Character	Older	0.98 (0.011)	0.97 (0.008)	0.99 (0.007)	0.99 (0.008)
	Young	0.96 (0.011)	0.96 (0.008)	0.97 (0.007)	0.96 (0.008)

*Standard errors appear in parentheses*.

### Reaction time

**Figure 1:**
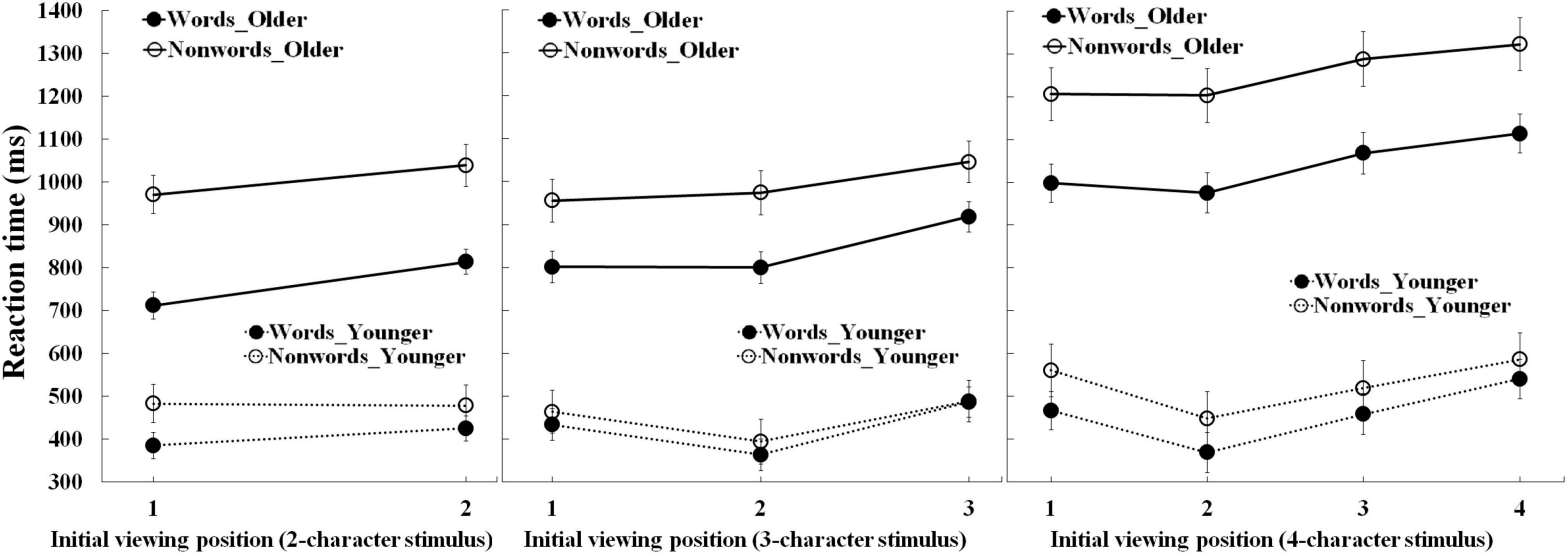
**Lexical decision times for the older and young adults as a function of the initial viewing position and stimulus type**. Bars correspond to standard errors.

Figure 1 shows the mean RTs for each stimulus length as a function of viewing position, stimulus type and age-group. Table 3 summarizes the statistical findings. For 2-, 3-, and 4-character stimulus length, there were main effects of age-group (*ps* < 0.001), due to shorter RTs for the young than older adults. Main effects of stimulus type (*ps* < 0.001) were due to shorter RTs for words than nonwords. Finally, there were main effects of viewing position (*ps* < 0.001). For the 2-character stimuli, RTs were shorter when the first rather than the second character was initially fixated. For 3- and 4-character stimuli, RTs were shortest when the second character was fixated initially. These results are in line with the findings by Liu and Li (2013).

**Table 3:** **Statistical values for analyses**.

	**Source of variance**	***F*-values**	***df***	***MSE***	**η*_p_*^2^**	***p***
**REACTION TIME**						
2-Char stimulus	Age × Position × Types	0.18	1, 46	2247	0.00	0.68
	Age × Position	7.76	1, 46	6981	0.14	0.008
	Age × Types	12.21	1, 46	27373	0.21	0.001
	Position × Types	8.50	1, 46	2247	0.16	0.005
	Age	82.37	1, 46	113548	0.64	< 0.001
	Types	44.17	1, 46	27373	0.49	< 0.001
	Position	18.06	1, 46	6981	0.28	< 0.001
3-Char stimulus	Age × Position × Types	0.27	2, 92	2472	0.01	0.77
	Age × Position	7.16	2, 92	5893	0.14	0.001
	Age × Types	9.41	1, 46	33126	0.17	0.004
	Position × Types	3.84	2, 92	2472	0.08	0.025
	Age	80.50	1, 46	216332	0.64	< 0.001
	Types	16.29	1, 46	33126	0.26	< 0.001
	Position	44.19	2, 92	5893	0.49	< 0.001
4-Char stimulus	Age × Position × Types	1.11	3, 138	2580	0.02	0.35
	Age × Position	8.29	3, 138	6054	0.15	< 0.001
	Age × Types	6.99	1, 46	73368	0.13	0.011
	Position × Types	1.30	3, 138	2580	0.03	0.28
	Age	80.50	1, 46	216332	0.64	< 0.001
	Types	26.83	1, 46	73368	0.37	< 0.001
	Position	55.31	3, 138	6054	0.55	< 0.001
**REFIXATION PROBABILITY**						
2-Char stimulus	Age × Position × Types	4.69	1, 46	0.006	0.09	0.036
	Age × Position	4.37	1, 46	0.019	0.09	0.042
	Age × Types	0.04	1, 46	0.013	0.00	0.85
	Position × Types	2.32	1, 46	0.006	0.05	0.13
	Age	119.08	1, 46	0.12	0.72	< 0.001
	Types	33.69	1, 46	0.013	0.42	< 0.001
	Position	7.51	1, 46	0.019	0.14	0.009
3-Char stimulus	Age × Position × Types	1.10	2, 92	0.005	0.02	0.34
	Age × Position	2.21	2, 92	0.020	0.05	0.12
	Age × Types	1.54	1, 46	0.010	0.03	0.22
	Position × Types	7.73	2, 92	0.005	0.14	0.001
	Age	108.55	1, 46	0.18	0.70	< 0.001
	Types	4.69	1, 46	0.010	0.09	0.036
	Position	4.59	2, 92	0.020	0.09	0.013
4-Char stimulus	Age × Position × Types	2.60	3, 138	0.004	0.05	0.055
	Age × Position	13.68	3, 138	0.011	0.23	< 0.001
	Age × Types	9.64	1, 46	0.011	0.17	0.003
	Position × Types	4.23	3, 138	0.004	0.08	0.007
	Age	72.92	1, 46	0.22	0.61	< 0.001
	Types	9.77	1, 46	0.011	0.18	0.003
	Position	18.05	3, 138	0.011	0.28	< 0.001
**FIRST FIXATION DURATION**						
2-Char stimulus	Age × Position × Types	1.34	1, 46	1123	0.03	0.25
	Age × Position	8.71	1, 46	3019	0.16	0.005
	Age × Types	2.08	1, 46	825	0.04	0.16
	Position × Types	2.29	1, 46	1123	0.05	0.14
	Age	14.46	1, 46	10811	0.24	< 0.001
	Types	46.14	1, 46	825	0.50	< 0.001
	Position	45.49	1, 46	3019	0.50	< 0.001
3-Char stimulus	Age × Position × Types	1.55	2, 92	654	0.03	0.22
	Age × Position	28.80	2, 92	2790	0.39	< 0.001
	Age × Types	11.54	1, 46	914	0.20	0.001
	Position × Types	3.54	2, 92	654	0.07	0.033
	Age	0.47	1, 46	10961	0.01	0.50
	Types	10.46	1, 46	914	0.19	0.002
	Position	9.64	2, 92	2790	0.17	< 0.001
4-Char stimulus	Age × Position × Types	1.92	3, 138	473	0.04	0.13
	Age × Position	18.24	3, 138	3541	0.28	< 0.001
	Age × Types	0.02	1, 46	697	0.00	0.89
	Position × Types	0.43	3, 138	473	0.01	0.73
	Age	0.41	1, 46	10202	0.01	0.53
	Types	20.40	1, 46	697	0.31	< 0.001
	Position	5.44	3, 138	3541	0.11	0.001

*Char, Character. p-values were Bonferroni-adjusted*.

While there were no three-way interactions, significant two-way interactions between age-group and viewing position for each stimulus length suggested age differences in OVP effects. For the 2-character stimuli, this interaction was due to a larger advantage of initially fixating the first than second character of each stimulus for the older adults (85 ms effect) than the young adults (18 ms effect). For the 3- and 4-character stimuli (see Tables 4, 5), RTs were equally shorter for the older adults when initially fixating the first or second characters than other character positions. But for the young adults, RTs for the 3- and 4-character stimuli were shortest when viewing the second character. Additionally, as shown in Table 6, polynomial trend analyses showed that for the young adults all trends were reliable for the 3-character (both linear and quadratic, *ps* < 0.05) and 4-character stimuli (linear, quadratic, and cubic, *ps* < 0.01). For older adults, only the linear trend was significant for 3-character nonwords (*p* = 0.001) and 4-character stimuli (*ps* < 0.001), though both linear and quadratic trends were reliable for 3-character words (*ps* < 0.01). Both the ANOVA and trend results indicated that RTs for the 3-character nonwords and 4-character stimuli exhibited reliable curves as a function of the initial viewing position with a minimum toward the first character for older adults, and the second character for young adults. Thus, the indication is that there are subtle but potentially important adult age differences in the OVP effect in Chinese.

**Table 4:** **Reaction time and eye movement measures: results of ANOVA and the pairwise comparisons between different imposed initial viewing positions for the 3-character stimuli for the older and young adults**.

		**Reaction time**				**Refixation probability**				**First fixation duration**			
		***F***	***MSE***	**η${}_{p}^{2}$**	***p***	***F***	***MSE***	**η*_*p*_*^2^**	***p***	***F***	***MSE***	**η*_*p*_*^2^**	***p***
**WORDS**													
	Older	14.09	7906	0.38	< 0.001	8.29	0.009	0.27	0.001	0.45	1646	0.02	0.64
	Young	56.09	1625	0.71	< 0.001	5.07	0.015	0.18	0.01	48.06	1140	0.68	< 0.001
Char 1 vs. Char 2	Older	0.001	9776	0.00	0.97	4.57	0.024	0.17	0.04				
	Young	35.26	3302	0.61	< 0.001	0.88	0.039	0.04	0.36	39.33	2475	0.63	< 0.001
Char 1 vs. Char 3	Older	12.76	26044	0.36	0.002	10.57	0.028	0.32	0.004				
	Young	16.73	4063	0.42	< 0.001	4.62	0.028	0.17	0.042	12.46	1717	0.35	0.002
Char 2 vs. Char 3	Older	28.92	11617	0.56	< 0.001	18.18	0.003	0.44	< 0.001				
	Young	152.11	2382	0.87	< 0.001	12.21	0.025	0.35	0.002	79.27	2650	0.78	< 0.001
**NONWORDS**													
	Older	9.82	5502	0.30	< 0.001	4.23	0.003	0.16	0.021	3.54	3081	0.13	0.037
	Young	33.13	1698	0.59	< 0.001	0.41	0.021	0.02	0.66	43.22	1022	0.65	< 0.001
Char 1 vs. Char 2	Older	0.70	11931	0.03	0.41	2.04	0.009	0.08	0.17	0.60	5243	0.03	0.45
	Young	31.91	3592	0.58	< 0.001					27.11	2604	0.54	< 0.001
Char 1 vs. Char 3	Older	14.19	13677	0.38	0.001	8.43	0.006	0.27	0.008	4.91	4344	0.18	0.037
	Young	4.75	2953	0.17	0.04					13.78	1614	0.38	0.001
Char 2 vs. Char 3	Older	16.47	7406	0.42	< 0.001	2.69	0.002	0.11	0.12	4.59	8897	0.17	0.043
	Young	57.35	3641	0.71	< 0.001					89.97	1913	0.80	< 0.001

*For all measures, the degrees of freedom were (2, 46) for the main effect of each age group and (1, 23) for the pairwise comparison. Char, character. p-values were Bonferroni-adjusted*.

**Table 5:** **Reaction time and eye movement measures: results of ANOVA and the pairwise comparisons between different imposed initial viewing positions for the 4-character stimuli for the older and young adults**.

		**Reaction time**				**Refixation probability**				**First fixation duration**			
		***F***	***MSE***	**η*_*p*_*^2^**	***p***	***F***	***MSE***	**η*_*p*_*^2^**	***p***	***F***	***MSE***	**η*_*p*_*^2^**	***p***
**WORDS**													
	Older	13.10	7440	0.36	< 0.001	3.22	0.002	0.12	0.028	6.81	2496	0.23	< 0.001
	Young	57.23	2090	0.71	< 0.001	14.27	0.017	0.38	< 0.001	20.44	1310	0.47	< 0.001
Char 1 vs. Char 2	Older	0.45	26436	0.02	0.51	0.22	0.002	0.01	0.65	0.77	2127	0.03	0.39
	Young	59.48	3362	0.75	< 0.001	20.58	0.039	0.47	< 0.001	14.99	2335	0.40	0.001
Char 1 vs. Char 3	Older	10.85	10951	0.32	0.003	4.08	0.004	0.15	0.055	0.044	3094	0.00	0.84
	Young	0.40	3926	0.02	0.54	0.34	0.0400	0.01	0.57	2.07	2555	0.08	0.16
Char 1 vs. Char 4	Older	46.48	6974	0.67	< 0.001	4.09	0.004	0.15	0.055	10.15	7001	0.31	0.004
	Young	34.21	3807	0.60	< 0.001	1.88	0.035	0.08	0.18	23.22	1813	0.50	< 0.001
Char 2 vs. Char 3	Older	10.99	18690	0.32	0.003	3.07	0.004	0.12	0.093	0.69	3923	0.03	0.41
	Young	33.33	5912	0.59	< 0.001	19.34	0.031	0.46	< 0.001	23.26	2902	0.50	< 0.001
Char 2 vs. Char 4	Older	25.04	18350	0.52	< 0.001	3.62	0.007	0.14	0.07	5.93	8610	0.21	0.023
	Young	132.09	5396	0.85	< 0.001	32.18	0.041	0.58	< 0.001	47.22	3259	0.67	< 0.001
Char 3 vs. Char 4	Older	6.41	7879	0.22	0.019	0.096	0.001	0.00	0.76	14.88	5201	0.39	0.001
	Young	59.88	2677	0.72	< 0.001	7.06	0.020	0.24	0.014	6.14	2857	0.21	0.021
**NONWORDS**													
	Older	15.60	5552	0.40	< 0.001	2.44	0.001	0.10	0.072	5.63	2549	0.20	0.002
	Young	40.05	2187	0.64	< 0.001	12.78	0.01	0.36	< 0.001	16.05	1672	0.41	< 0.001
Char 1 vs. Char 2	Older	0.018	14106	0.00	0.89					0.027	3203	0.00	0.87
	Young	71.03	4244	0.76	< 0.001	22.37	0.031	0.49	< 0.001	20.33	3457	0.47	< 0.001
Char 1 vs. Char 3	Older	26.89	6032	0.54	< 0.001					0.049	3707	0.00	0.83
	Young	13.75	2925	0.37	0.001	10.83	0.018	0.32	0.003	0.59	2925	0.03	0.45
Char 1 vs. Char 4	Older	44.62	7330	0.66	< 0.001					8.49	6643	0.27	0.008
	Young	10.51	1612	0.31	0.004	1.10	0.024	0.04	0.31	4.84	2441	0.17	0.036
Char 2 vs. Char 3	Older	11.38	15411	0.33	0.003					0.11	4880	0.00	0.75
	Young	13.30	9131	0.37	0.001	6.74	0.023	0.23	0.016	31.64	2975	0.58	< 0.001
Char 2 vs. Char 4	Older	23.76	14545	0.51	< 0.001					6.44	8077	0.22	0.018
	Young	65.95	6996	0.74	< 0.001	24.86	0.018	0.52	< 0.001	33.42	4181	0.59	< 0.001
Char 3 vs. Char 4	Older	3.11	9194	0.12	0.09					15.46	4074	0.40	0.001
	Young	82.18	1331	0.78	< 0.001	7.07	0.011	0.24	0.014	1.10	4089	0.05	0.31

*For all measures, the degrees of freedom were (3, 69) for the main effect of each age group and (1, 23) for the pairwise comparison. Char, character. p-values were Bonferroni-adjusted*.

**Table 6:** **Polynomial trend analyses of RTs and eye movement measures for the 3- and 4-character stimuli for the older and young adults**.

	**Reaction time**				**Refixation probability**				**First fixation duration**			
	***F***	***MSE***	**η*_*p*_*^2^**	***p***	***F***	***MSE***	**η*_*p*_*^2^**	***p***	***F***	***MSE***	**η*_*p*_*^2^**	***p***
**OLDER__3-CHARACTER WORD**												
Linear	12.76	13022	0.36	0.002	10.57	0.014	0.32	0.004	0.65	886	0.027	0.43
Quadratic	20.29	2790	0.47	< 0.001	0.55	0.004	0.023	0.47	0.38	2405	0.016	0.55
**YOUNG__3-CHARACTER WORD**												
Linear	16.73	2032	0.42	< 0.001	4.62	0.014	0.17	0.042	12.46	858	0.35	0.002
Quadratic	121.76	1218	0.84	< 0.001	5.46	0.016	0.19	0.029	69.54	1422	0.75	< 0.001
**OLDER__3-CHARACTER NONWORD**												
Linear	14.19	6839	0.38	0.001	8.43	0.003	0.27	0.008	4.91	2172	0.18	0.037
Quadratic	2.66	4166	0.10	0.12	0.17	0.003	0.007	0.68	2.79	3989	0.11	0.11
**YOUNG__3-CHARACTER NONWORD**												
Linear	4.75	1477	0.17	0.040	0.045	0.018	0.002	0.84	4.75	1477	0.17	0.04
Quadratic	54.97	1919	0.71	< 0.001	0.69	0.024	0.029	0.42	54.97	1919	0.71	< 0.001
**OLDER__4-CHARACTER WORD**												
Linear	70.33	3320	0.75	< 0.001	4.33	0.004	0.16	0.049	6.98	4003	0.23	0.015
Quadratic	2.92	9488	0.11	0.10	0.46	0.000	0.019	0.51	6.97	2027	0.23	0.015
Cubic	3.28	9511	0.13	0.083	2.13	0.002	0.085	0.16	6.13	1459	0.21	0.021
**YOUNG__4-CHARACTER WORD**												
Linear	46.59	2501	0.67	< 0.001	5.76	0.021	0.20	0.025	36.34	1054	0.61	< 0.001
Quadratic	138.41	1410	0.86	< 0.001	21.96	0.018	0.49	< 0.001	16.23	1572	0.41	0.001
Cubic	19.98	2358	0.47	< 0.001	17.26	0.012	0.43	< 0.001	12.65	1303	0.36	0.002
**OLDER__4-CHARACTER NONWORD**												
Linear	62.23	3660	0.73	< 0.001	5.30	0.001	0.19	0.031	6.08	3909	0.21	0.022
Quadratic	1.62	5284	0.066	0.22	0.24	0.001	0.010	0.63	7.75	1885	0.25	0.011
Cubic	3.04	7710	0.12	0.095	1.15	0.001	0.048	0.30	2.53	1852	0.099	0.13
**YOUNG__4-CHARACTER NONWORD**												
Linear	14.89	1834	0.39	0.001	0.040	0.011	0.002	0.84	14.89	1834	0.39	0.001
Quadratic	162.87	1188	0.88	< 0.001	39.73	0.008	0.63	< 0.001	162.87	1188	0.88	< 0.001
Cubic	11.84	3538	0.34	0.002	7.25	0.012	0.24	0.013	11.84	3538	0.34	0.002

*For all measures, the degrees of freedom were (1, 23) for these polynomial trend analyses. p-values were Bonferroni-adjusted*.

Additional interactions between age-group and stimulus type for each stimulus length were due to larger increases in RTs for nonwords than words for the older than young adults (*ps* < 0.05), and interactions between viewing position and stimulus type for the 2- and 3-character stimuli were due to a larger viewing position effect for words than nonwords (*ps* < 0.05).

### Refixation probability

**Figure 2:**
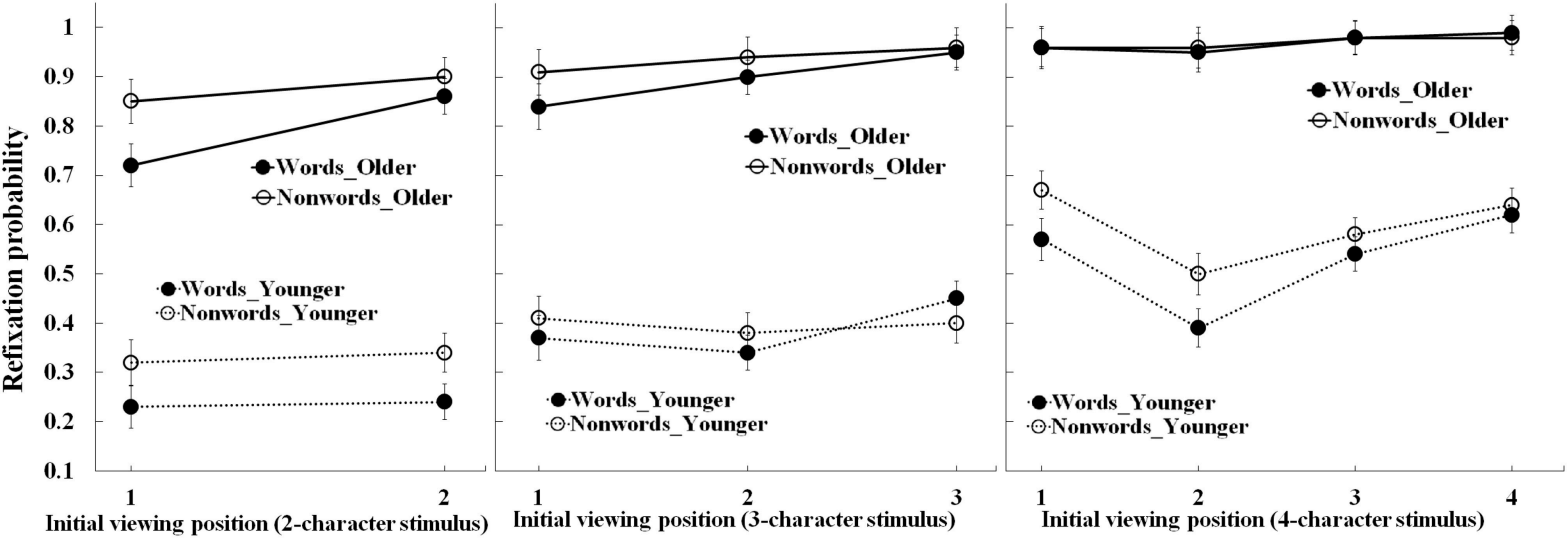
**Refixation probability for the older and young adults as a function of the initial viewing position and stimulus type**. Bars correspond to standard errors.

Figure 2 shows refixation probabilities for 2-, 3-, and 4-character stimuli as a function of viewing position, stimulus type, and age-group. Table 3 summarizes the statistical findings. For each stimulus length, there were main effects of age-group, due to fewer refixations for the young than older adults (*ps* < 0.001). There were also main effects of stimulus type (*ps* < 0.05) due to lower refixation rates for words than nonwords, and main effects of viewing position (*ps* < 0.05). For the 2-character stimuli, refixation probability was lower when the first rather than second character was initially fixated (*p* < 0.01). For the 3-character stimuli, refixation probability was equally lowest when the first or second character was initially fixated (*ps* < 0.01). Finally, for the 4-character stimuli, refixation probability was lower when the second character was viewed initially (*ps* < 0.001). However, these effects were qualified by several interactions.

In particular, there were two-way interactions between age-group and viewing position for the 2- and 4-character stimuli, qualified for the 2-character stimuli by a three-way interaction that included stimulus type. For the 2-character stimuli, the two-way interaction was due to a lower refixation rate for the older adults than the young adults when the first character rather than the second character was viewed initially. The three-way interaction revealed this effect was larger for words. For the 4-character stimuli (see Figure 2 and Table 5), the interaction between age-group and viewing position was due to a lower refixation probability when the second character was initially fixated by the young adults than the older adults. As shown in Table 6, polynomial trend analyses for the older adults showed that only linear trends were reliable for the 3- and 4-character stimuli (*ps* < 0.05), which indicated that the refixation probability was lowest when the initial viewing position was the first character. For the young adults, these analyses showed that all trends were reliable for the 3-character words (both linear and quadratic, *ps* < 0.05) and 4-character words (linear, quadratic and cubic, *ps* < 0.05), which indicated that refixation probability as a function of initial viewing position exhibited a U-shaped curve with a minimum toward the second character. Thus, these analyses confirmed differences in the influence of fixation position on word recognition for young and older adults.

For the 3- and 4-character stimuli, additional interactions between viewing position and stimulus type were due to larger effects of viewing position (when the first rather than the last character was initially fixated) on refixation rates for words than nonwords. Meanwhile, an interaction between age-group and stimulus type for the 4-character stimuli was due to the fewer refixations on words than nonwords by young adults (*p* = 0.003), but not older adults (*p* = 0.97).

### First fixation duration

**Figure 3:**
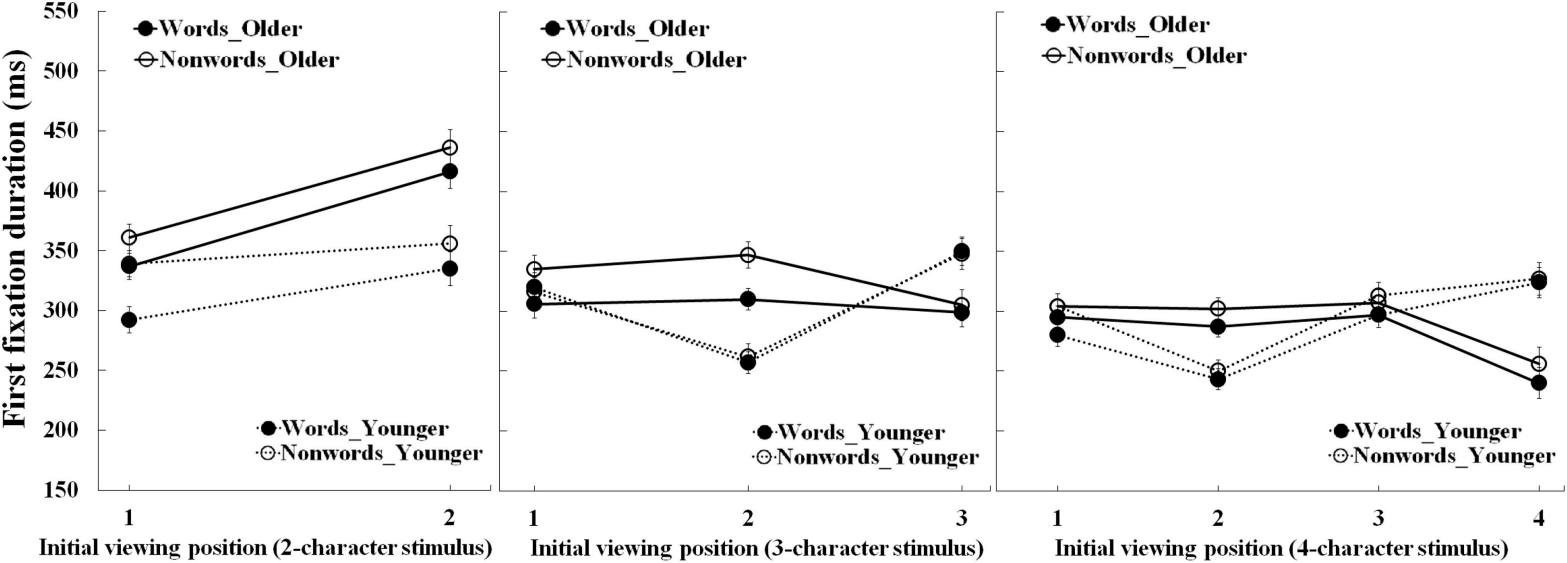
**First fixation duration for the older and young adults as a function of the initial viewing position and stimulus type**. Bars correspond to standard errors.

Figure 3 shows the first fixation durations for 2-, 3-, and 4-character stimuli as a function of viewing position, stimulus type and age-group. Table 3 summarizes the statistical findings. There was a main effect of age-group for the 2-character stimuli only (*p* < 0.001), due to shorter fixations by the young than older adults. There were also main effects of stimulus type for each stimulus length (*ps* < 0.01), due to shorter fixations for words than nonwords. Finally, there were main effects of viewing position for each stimulus length (*ps* < 0.01). For the 2-character stimuli, this was due to shorter fixations when the first rather than the second character was fixated initially. For the 3- and 4-character stimuli, the effect was due to fixations being shortest when the second characters were fixated initially. These effects were qualified by two-way interactions between age-group and viewing position.

For the 2-character stimuli, this interaction was due to a larger decrease in fixation duration when the first rather than second character was initially fixated by the older adults (77 ms, *p* < 0.001) compared to the young adults (30 ms, *p* = 0.005). For the 3- and 4-character stimuli, the interaction was due to different patterns of viewing position effect for the young and older adults. For the older adults, fixations were shortest when the initial viewing position was the final character of these stimuli (see Tables 4, 5). As shown in Table 6, for the older adults, polynomial trend analyses showed that only the linear trend was reliable for 3-character nonwords (*p* = 0.037), and both linear and quadratic trends were reliable for 4-character stimuli (*ps* < 0.05). These results indicated that older adults initially produced shorter fixations when the initial viewing position was the final character, most likely because this was a suboptimal viewing position for these longer words. As a result, fixations may have been curtailed by the rapid initiation of a saccade to refixation at a more optimal location in the words. By contrast, for the young adults, fixations were shortest for the 3- and 4-character stimuli when the initial viewing position was the second character. Polynomial trend analyses for the young adults showed that reliable linear and quadratic trends for 3- and 4-character stimuli (*ps* < 0.05), and reliable cubic curves for 4-character stimuli (*ps* < 0.01). These indicated that fixations were shortest when the initial viewing position was the second character of 3- and 4-character stimuli for young adults. In short, these effects suggest a stronger effect of initial viewing position for the young than older adults for the longer stimuli.

## General discussion

This study is the first to examine adult age differences in the OVP effect in Chinese word recognition. A particularly comprehensive and informative measure of word recognition efficiency is the time taken to make a lexical decision. In the present experiment, this revealed clear effect of fixation location on word recognition for both young and older adults (see Table 7). In particular, reaction times for young adults were broadly in line with findings from the only previous investigation of the OVP effect in isolated Chinese word recognition (Liu and Li, 2013). Moreover, as broadly similar effects of fixation location on information efficiency (i.e., RT) were obtained for word and nonword stimuli, the benefit of fixating a specific character location in a Chinese character-string appears to reflect the normal oculomotor strategy of these readers rather than a specific advantage for word recognition.

**Table 7:** **Summary of OVP patterns due to ANOVA and polynomial trend analyses of RTs and eye movement measures for the older and young adults**.

		**Reaction time**		**Refixation probability**		**First fixation duration**	
		**ANOVA**	**Trend**	**ANOVA**	**Trend**	**ANOVA**	**Trend**
2-Char words	Older	➀		➀		➀	
	Young	➀		X		➀	
2-Char nonwords	Older	➀		➀		➀	
	Young	X		X		X	
3-Char words	Older	➀≈➁	➁	➀	➀	X	X
	Young	➁	➁	➀≈➁	➁	➁	➁
3-Char nonwords	Older	➀≈➁	➀	➀≈➁	➀	➂	➂
	Young	➁	➁	X	X	➁	➁
4-Char words	Older	➀≈➁	➀	X	➀	➃	➃
	Young	➁	➁	➁	➁	➁	➁
4-Char nonwords	Older	➀≈➁	➀	X	➀	➃	➃
	Young	➁	➁	➁	➁	➁	➁

*X, no OVP for this measure. ➀, ➁, ➂, and ➃indicate that there could be OVP patterns when the initial viewing positions were these characters, respectively. ➀≈➁ indicates that there could be OVP patterns when the initial viewing positions were the first or second characters*.

Crucially, however, the reaction time data revealed subtle but potentially important adult age differences in this OVP effect. The older adults were slower to recognize words (and nonwords) than the young adults, consistent with findings from previous research in alphabetic languages showing that older adults are generally slower to make lexical decisions (Balota et al., 2004; Ratcliff et al., 2004; Stine-Morrow et al., 2006). Importantly, the present findings in addition revealed adult age differences in the effects of fixation location. Compared to the young adults, the older adults showed a larger advantage of initially fixating the first rather than the second character of 2-character stimuli. Additionally, by contrast with the young adults, the older adults recognized 3- and 4-character stimuli most efficiently when they initially fixated the first rather than the second character of these stimuli (see Table 7). Therefore, the older adults' recognition of words (and nonwords) showed a much stronger benefit of initially fixating the beginning character of Chinese character-strings. This was unlikely to be a consequence of the older adults being generally poorer at recognizing words, as both age-groups produced high rates of word recognition accuracy (>96%). Moreover, the similarity in the pattern of the effects of fixation location on reaction times for words and nonwords suggests this was not a specific influence on word recognition but more likely reflected adult age differences in the oculomotor strategy used to process of character-strings.

Further indications of an age difference in oculomotor behavior came from the eye movement data. Measures of eye movements were used to ensure that participants accurately fixated designated fixation locations in the stimuli (e.g., Brysbaert and Nazir, 2005; Jordan and Paterson, 2009; Yao-N'Dre et al., 2013), but provide additional insights into the influence of fixation location on word recognition. Compared to the young adults, the older adults made more refixations on character-strings of each length, and longer first fixations on the 2-character stimuli. The eye movement data were therefore broadly consistent with evidence from the reaction time data indicating that older adults found word recognition more effortful. The older adults also showed a larger reduction in refixation rate and first fixation duration, compared to the young adults, when they fixated the first rather than second character of 2-character stimuli, again in line with evidence from the reaction time data showing the older adults benefited more from fixating the first character of these stimuli.

For the 3- and 4-character stimuli, the data provide a clearer indication of an OVP effect in the eye movement behavior of the young adults than older adults. For 3-character words, the older adults had a lower refixation rate when they initially fixated the first character than any other character. But, no effect of fixation location on the length of their first fixations was observed. For 4-character words, no effect of fixation location on refixation rates was observed for the older adults and first fixations were shorter only when the older adults initially fixated the final character of these stimuli. The indication, therefore, is that the older adults experienced particular difficulty recognizing the longer character-strings. Moreover, the older adults may have made shorter fixations when initially fixating the final character of these stimuli because this was an especially suboptimal fixation location and they may have rapidly made a corrective saccade to shift their gaze to a more optimal location. The young adults did not show this reduction in fixation time when they initially fixated the final character of 3- and 4-character strings. Consequently, the effect observed for the older adults may reflect their general poorer acuity, especially in more peripheral vision. By comparison, for the young adults, refixation probabilities were lowest and first fixations shortest when the second character was initially viewed for both the 3- and 4-character words. This pattern was broadly in line with the indication from the reaction time data (and previous findings by Liu and Li, 2013) that word recognition is optimal for young adults when they initially fixate the second character of 3- and 4-character stimuli. Previous research has shown a clear inverted optimal viewing position (IOVP) effect in the duration of the first fixation on words (Vitu et al., 2001, 2007; Nuthmann et al., 2005), such that fixations are longer at the word center and shorter at the word boundaries. This IOVP effect was not clearly visible in the present experiment, quite possibly as a consequence of the very different procedure we used, which required readers to fixate a designated location until the stimulus appeared. However, the effects we observed nevertheless support the view that older adults produce a qualitatively different pattern of oculomotor behavior compared to young adults when recognizing multi-character Chinese words presented in isolation.

These patterns of eye movement behavior may well-reflect important differences in the processing capabilities of the two age-groups. In particular, it is well-established that visual and cognitive declines in later life affect older adults' processing of linguistic stimuli (Rayner et al., 2006; Stine-Morrow et al., 2006). Moreover, older adults may have a smaller and less asymmetric perceptual span than young adults and this may place further limits on their information processing (Rayner et al., 2009). The visual and cognitive declines experienced in later life are very likely to contribute to findings that older adults process text more slowly and make more and longer fixations on words ( e.g., Rayner et al., 2010, 2014; Risse and Kliegl, 2011; Kuperman and van Dyke, 2011; Payne and Stine-Morrow, 2012; Paterson et al., 2013; Payne et al., 2014). Moreover, it would be unsurprising to find that these factors also underlie the adult age differences in word recognition performance in the present experiment. In particular, similarly to these previous studies, we found older adults responded more slowly and made more and longer fixations compared to young adults when recognizing words. Nevertheless, the extent to which visual and cognitive declines contribute to adult age differences in the effects of fixation location on word recognition remains to be fully determined, although it may be that this led the older adults in the present experiment to employ a more careful oculomotor strategy. Indeed, one possibility is the older adults use an especially careful strategy during Chinese word recognition in which they initially fixate the first character in a character-string and make multiple fixations on this string to identify the word.

These findings may also reflect more general adult age differences in oculomotor behavior when reading Chinese. While the precise nature of eye-guidance during Chinese reading remains controversial (e.g., Yang and McConkie, 1999; Tsai and McConkie, 2003; Yan et al., 2010; Li et al., 2011, 2015), it is increasingly accepted that words are not systematically selected as saccade targets, and that the targeting of saccades reflects the extent to which readers can identify upcoming characters in a sentence. Indeed, Yan et al. showed that readers have a strong tendency to fixate the center of a multi-character word when this word receives only a single fixation but are more likely to fixate the beginning of a word when it receives multiple fixations. Yan et al. took this as evidence for flexibility in saccade-target selection and that readers select the beginning or center of words as targets depending on their success in processing upcoming character information. The degree to which older adults select the beginning or center of words as saccade targets is yet to be determined and, in general, researchers have yet to provide a detailed account of adult age differences in eye movement control during Chinese reading. Consequently, it also remains to be determined if the subtle age differences in the OVP effect observed in the present research resonate with adult age differences in eye movement control during natural reading. Clearly it will be important for future research to address this question and, in particular, to establish if older Chinese readers employ a more careful oculomotor strategy. Moreover, despite the wealth of research on the OVP effect in alphabetic languages, to our knowledge there are no published studies on the effects of normal aging on the OVP effect in these languages. Given the very different construction of words in alphabetic and logographic languages, it is not obvious if effects of fixation location on word recognition in alphabetic languages will be similar to those observed in the present study. But it will nevertheless be valuable for future research to examine whether such adult age differences in the OVP effect are observed, as this will shed further light on the nature of the difficulties experienced by older readers.

In sum, the present study provides further evidence of an OVP effect during the recognition of isolated Chinese words and novel insights into adult age differences in the influence of fixation location on word recognition in Chinese. The findings suggest that, compared to young adults, older adults have particular difficulty recognizing Chinese words, especially longer words, and employ a more cautious strategy in which they initially fixate the beginning character of words and make multiple fixations on character-strings during word recognition. Whether, this more cautious reading strategy is a general characteristic of the oculomotor behavior of older Chinese readers remains to be determined. Nevertheless, these age-related differences in the OVP effect may make an important contribution to understanding how older Chinese readers adapt their saccade-targeting strategies to compensate for visual and cognitive declines.

### Conflict of interest statement

The authors declare that the research was conducted in the absence of any commercial or financial relationships that could be construed as a potential conflict of interest.
